# Effect of childhood vaccination and antibiotic use on pneumococcal populations and genome-wide associations with disease among children in Nepal: an observational study

**DOI:** 10.1016/S2666-5247(22)00066-0

**Published:** 2022-07

**Authors:** Rama Kandasamy, Stephanie Lo, Meeru Gurung, Michael J Carter, Rebecca Gladstone, John Lees, Sonu Shrestha, Stephen Thorson, Sanjeev Bijukchhe, Madhav C Gautam, Reetu Shrestha, Sunaina Gurung, Bibek Khadka, Lesley McGee, Robert F Breiman, David R Murdoch, Dominic F Kelly, Shrijana Shrestha, Stephen D Bentley, Andrew J Pollard

**Affiliations:** a School of Women and Children's Health, University of New South Wales, Sydney, NSW, Australia; b Oxford Vaccine Group, Department of Paediatrics, University of Oxford, Oxford, UK; c NIHR Oxford Biomedical Research Centre, Oxford, UK; d Parasites and Microbes Programme, Wellcome Sanger Institute, Hinxton, Cambridgeshire, UK; e European Molecular Biology Laboratory, European Bioinformatics Institute EMBL-EBI, Hinxton, UK; f Paediatric Research Unit, Patan Academy of Health Sciences, Kathmandu, Nepal; g Department of Women and Children's Health, School of Life Course Sciences, King's College London, London, UK; h Respiratory Diseases Branch, Centers for Disease Control and Prevention, Atlanta, GA, USA; i Department of Global Health, Rollins School of Public Health, Emory University, Atlanta, GA, USA; j Department of Pathology, University of Otago, Christchurch, New Zealand

## Abstract

**Background:**

Pneumococcal disease is a leading cause of bacterial pneumonia and invasive bacterial disease among children globally. The reason some strains of pneumococci are more likely to cause disease, and how interventions such as vaccines and antibiotics affect pneumococcal strains is poorly understood. We aimed to identify genetic regions under selective pressure and those associated with disease through the analysis of pneumococcal whole-genome sequences.

**Methods:**

Whole-genome sequencing was performed on pneumococcal isolates collected between January, 2005, and May, 2018, in Kathmandu, Nepal, which included programmatic ten-valent pneumococcal conjugate vaccine (PCV10) introduction in 2015. Isolates were from three distinct cohorts: nasopharyngeal swabs of healthy community-based children, nasopharyngeal swabs of children admitted to hospital with pneumonia, and sterile-site cultures from children admitted to hospital. Across these cohorts we examined serotype distribution, antibiotic resistance, strain distribution, and regions of recombination to determine genes that were undergoing diversifying selection. Genome-wide association studies comparing pneumonia and sterile-site isolates with healthy carriage were used to determine novel variants associated with disease.

**Findings:**

After programmatic introduction of PCV10, there was a decline in vaccine covered serotypes; however, strains that had expressed these serotypes continued to exist in the post-PCV population. We identified GPSC9 to be a strain of concern due to its high prevalence in disease, multidrug resistance, and ability to switch to an unencapsulated phenotype via insertion of virulence factor *pspC* into the cps locus. Antibiotic resistance loci to co-trimoxazole were found to be prevalent (pre-PCV10 78% *vs* post-PCV10 81%; p=0·27) and increasingly prevalent to penicillin (pre-PCV10 15% *vs* post-PCV10 32%; p<0·0001). Regions with multiple recombinations were identified spanning the surface protein virulence factors *pspA* and *pspC* and antibiotic targets *pbpX, folA, folC, folE*, and *folP*. Furthermore, we identified variants in *lacE2* to be strongly associated with isolates from children with pneumonia and *PRIP* to be strongly associated with isolates from sterile sites.

**Interpretation:**

Our work highlights the effect of pneumococcal conjugate vaccines, antibiotics, and host–pathogen interaction in pneumococcal variation, and the pathogen's capability of adapting to these factors at both population-wide and strain-specific levels. Ongoing surveillance of disease-associated strains and further investigation of *lacE2* and *PRIP* as serotype-independent targets for therapeutic interventions is required.

**Funding:**

Gavi, The Vaccine Alliance; WHO; Bill & Melinda Gates Foundation; Wellcome Sanger Institute; and US Centers for Disease Control and Prevention.

## Introduction

The widespread use of antibiotics and adoption of pneumococcal conjugate vaccines (PCVs) in infant immunisation programmes globally have reduced childhood morbidity and mortality due to *Streptococcus pneumoniae*; however, pneumococcal disease continues to be the largest cause of bacterial pneumonia and meningitis among children, with estimates indicating it is currently responsible for 300 000 childhood deaths globally each year.^1^ Additionally, the progressive rise of antibiotic resistance and the increasing incidence of invasive pneumococcal disease due to non-vaccine serotypes in settings with mature PCV programmes^2^ highlights the need to better understand the evolutionary adaptations of pneumococci after introduction of these clinical interventions.^3^

Studying the molecular epidemiology of pneumococcal populations using whole-genome sequencing is a highly informative approach to understanding the effects of clinical interventions on pneumococcus.^4^ Early studies have achieved this by using bioinformatic tools to identify and compare sequence variation between cohorts of pneumococci. For example, a study comparing cohorts of pneumococci collected from asymptomatic carriers during different time periods of PCV introduction showed a decline in the prevalence of serotypes covered by PCVs, but little effect on overall strain variation.^5^ Another study showed that non-encapsulated pneumococcal lineages, which are not targeted by PCVs, have a major role in adaptation via horizontal gene transfer.^6^

**Research in context**

**Evidence before this study**

We searched PubMed using the terms “pneumococcus” OR “*Streptococcus pneumoniae*” AND “vaccine” AND “sequencing”, for publications in English between Jan 1, 2000, and Oct 10, 2021. 171 results were reviewed with 24 publications meeting inclusion criteria. No studies had examined isolates from children with pneumonia. Two studies reported pneumococcal population genomics in Asia before vaccine introduction. One study from China (78 samples) had examined the effect of pneumococcal conjugate vaccines (PCVs) on pneumococcal strains, and only two studies that applied a genome-wide association analysis to disease isolates. One was a genome-wide association study (GWAS) of invasive disease-causing serotype 1 isolates, and the other was a GWAS of invasive disease isolates from adults, many of whom had underlying comorbidities. To our knowledge, the present study is the first to have analysed a consolidated pneumococcal population that spans healthy carriage, pneumonia, and invasive disease. We believe it also to be the largest study from Asia to report the effect of PCV use on pneumococcal molecular epidemiology and the first to report a GWAS on isolates collected from Asian children.

**Added value of this study**

This study examines a diverse collection of pneumococci from asymptomatic carriage and disease contexts to describe the effect of pneumococcal conjugate vaccination, antibiotic use, and host–pathogen interaction on the population genomics of pneumococcus in an Asian setting. The study identifies the GPSC9 lineage to be associated with a high proportion of pneumonia cases, multidrug resistant, capable of evading vaccine effect through recombination of the cps locus, and widely dispersed globally. This study also identifies novel pneumococcal genetic variants associated with disease using GWAS. The genes *lacE2* and *PRIP*, within which these variants of interest reside, necessitate further detailed study as potential targets for therapeutic interventions such as new antibiotics and vaccines which act independent of serotype.

**Implications of all the available evidence**

This study enhances the understanding of pneumococcal strain adaptation to changes in antibiotic and vaccine use in an Asian setting. Given that a large proportion of global childhood morbidity and mortality due to pneumococcus occurs in Asia, further work is urgently needed to improve the use of first-line antibiotics. Further investigation of the novel genetic variants associated with disease that were identified by this study provide one such possible avenue for future research.

Pneumococcal sequences might also be found to systematically differ between disease states, suggesting a differential advantage by the host niche. For example, a variant in the penicillin-binding protein gene *pbp1b* was found to be associated with isolates causing meningitis when compared with the sequences of non-meningitis-causing isolates.^7^ A study of serotype 1 pneumococcal isolates identified a choline-binding protein and a helicase protein to be associated with meningitis.^8^

We collected pneumococcal isolates from children residing within the Kathmandu Valley of Nepal over a 14-year period, which included the introduction of the ten-valent pneumococcal conjugate vaccine (PCV10) in August, 2015. This collection included isolates from children who were healthy carriers, children admitted to hospital with pneumonia, and children with invasive pneumococcal disease. Through the analyses of pneumococcal whole-genome sequences of this collection we aimed to better understand the effect of vaccine and antibiotic use on the pneumococcal population and how strains vary between healthy and disease states.

## Methods

### Study design and participants

Pneumococcal isolates were collected as part of paediatric (ie, children aged 0–14 years) pneumococcal carriage and disease surveillance studies conducted in Kathmandu, Nepal, between January, 2005, and May, 2018. A subset of these isolates were randomly selected, using a random number generator, for whole-genome sequencing from three distinct collection cohorts: nasopharyngeal swabs of healthy community-based children from the Kathmandu Valley, Nepal; nasopharyngeal swabs of children admitted to Patan Hospital (Lalitpur, Nepal), with pneumonia; and sterile site cultures (from pleural fluids, blood, and cerebrospinal fluid) of children admitted to Patan Hospital (appendix 1 pp 3–4). A subset of the children with pneumonia had detailed clinical data collected (appendix 1 p 5). From Aug 1, 2015, PCV10 (manufactured by GSK, Rixensart, Belgium) was included in the infant immunisation schedule at weeks 6 and 10, and 9 months. Isolates collected before and after Aug 1, 2015, were classified as pre-PCV and post-PCV cohorts, respectively. Ethical approval was obtained from the Oxford Tropical Research Ethics Committee (OXTREC 5908, 17–12, 28–14, 11–15, and 9–15) and the Nepal Health Research Council (registration numbers 31/2012, 39/2014, and 286/2014).

### Specimen collection and processing

Nasopharyngeal swabs were collected and processed by conventional microbiological methods according to WHO guidelines;^9^ ie, a single nylon flocked nasopharyngeal swab (Thermo Fisher Scientific, Leicester, UK) was collected from each participant, and placed in skim-milk-tryptone-glucose-glycerine medium. Swabs were streaked onto 5% sheep blood agar and the plates incubated at 37°C overnight. Normally sterile site isolates were identified after microbiological processing of clinical samples from children at Patan Hospital. Isolates were subcultured on 5% sheep blood agar before being stored at –70°C in Microbank bacterial storage tubes (Thermo Fisher Scientific, Leicester, UK). Phenotyping by Quellung reaction to reference serum samples (Serum Statens Institut, Copenhagen, Denmark) was performed on all isolates.

### Whole-genome sequencing

DNA was extracted using the DNeasy mini kit platform (Qiagen, Manchester, UK). All isolates were sequenced as part of the Global Pneumococcal Sequencing (GPS) project on the Wellcome Sanger Institute's core sequencing pipeline using Illumina HiSeq 4000 analysers with at least 75 base pair paired-end runs. Sample quality control is described in appendix 1 (p 2). Multilocus sequence types (MLSTs) were determined in silico using MLSTcheck (version 2.0.1510612) and serotype (capsular locus) using SeroBA (version 1.0.0).^10^, ^11^

### Mapping and alignment

Sequence reads of the selected isolates were mapped against the reference genome of *Streptococcus pneumoniae* ATCC 700669 (GenBank accession number NC_011900) using SMALT, version 0.7.6.^12^ Pseudogenomes generated using samtools mpileup were also aligned using *Streptococcus pneumoniae* ATCC 700669 as the reference. Consensus SNPs from the alignment were identified using single-nucleotide polymorphism (SNP) sites, version 2.3.2, and the SNP alignments of the full dataset and subsets (healthy carriers, pneumonia, and invasive pneumococcal disease) used to generate maximum-likelihood phylogenies using RaxML, version 8.2.8, with a generalised time-reversible model of nucleotide substitution with gamma model of rate heterogeneity.^13^, ^14^ Entire nucleotide sequence alignments of lactose-specific phosphotransferase system (PTS) gene *lacE2* and the putative replication initiator protein (*PRIP*) were used to generate maximum-likelihood phylogenies using RaxML. Sequence alignments of the capsular polysaccharide (cps) locus, and PRIP region were locally aligned using blastn and visualised using Easyfig, version 2.2.2.^15^, ^16^

### Clustering

Global Pneumococcal Sequencing Clusters (GPSCs) were determined using PopPUNK, version 2.2.0, as previously described.^4^ Other GPSC9 isolates from the GPS project were used to determine the global context of Nepalese GPSC9 isolates. Temporal signals within clusters were visualised and explored using TempEst, version 1.5.3.^17^ Regions of recombination within the ten most prevalent clusters were determined using the Gubbins algorithm on alignments against ATCC 700669.^18^ These data were then merged and hotspots of recombination across the entire ten clusters defined as regions where recombination frequency per base was in the top 5% across the genome.

### Antibiotic resistance loci

Whole-genome sequences for each isolate were screened for the presence of known antibiotic resistance loci in the US Centers for Disease Control and Prevention pneumococcal typing pipeline database^19^ and is described in appendix 1 (p 2). Because of the scarcity of published data, information on antibiotic use in Kathmandu, Nepal, was collected through interviews with two senior paediatricians who have worked at Patan Hospital, Kathmandu, for the previous two decades (appendix 1 p 6).

### Genome-wide association study

Detailed methods are described in the appendix 1 (p 2). Genome assemblies for isolates in each study cohort (pneumonia *vs* healthy carriers, and invasive pneumococcal disease *vs* healthy carriers) were used to inform analysis in ROARY^20^ before pneumococcal genome-wide association studies were conducted using a fixed-effects model of Clusters of Orthologous Groups of proteins and SNP association in pyseer.^21^ hierBAPS was applied to sequence alignments of *lacE2* and *PRIP* to determine gene-specific sequence clusters.^22^

### Statistical analysis

For antibiotic resistance pre-PCV10 and post-PCV10 introduction, we compared the proportion of susceptible with non-susceptible (intermediate or resistant) isolates. The difference in the proportion of antibiotic resistance between pre-PCV10 and post-PCV10 groups, proportion of PCV10-covered serotypes, and proportion of isolates with a deletion in *lacE2*, were compared using a one-sample Z test with continuity correction. For comparisons of proportions a p value of less than 0·05 was considered significant. For the genome-wide association studies, an indicative significant p value was determined by applying a Bonferroni adjustment for multiple testing at a p value of 0·01 with 31 708 tests for the pneumonia versus controls analysis and 82 942 tests for the invasive pneumococcal disease versus controls analysis. Unless otherwise stated, analyses were performed using R, version 4.0.3.

### Role of the funding source

The funders of the study had no role in study design, isolate selection, data analysis, data interpretation, or writing of the report. The corresponding author had full access to all the data in the study and had final responsibility for the decision to submit for publication.

## Results

935 pneumococcal isolates were included in the analyses—597 nasopharyngeal isolates from healthy children, 245 nasopharyngeal isolates from children admitted to hospital with pneumonia, and 93 isolates from normally sterile sites from children with invasive pneumococcal disease (appendix 1 p 3). 63 serotypes (n=874) plus non-typeable isolates (n=61) were identified. The five most common serotypes were 6A (n=63), 1 (n=61), non-typeable isolates (n=61), 23F (n=48) and 14 (n=43; appendix 1 p 7). Significantly more invasive pneumococcal disease isolates are covered by PCV10 (69 [74%] of 93) when compared with those from healthy children (116 [19%] of 597; p<0·0001) and children with pneumonia (86 [35%] of 245; p<0·0001; appendix 1 p 8).

PCV10 was introduced into the infant immunisation schedule in August, 2015. The distribution of sequenced serotypes reflected this clinical intervention with a decrease in proportion of PCV10 serotypes isolated when comparing isolates collected before (181 [36%] of 508) and after (83 [19%] of 427) this introduction (p<0·0001). When comparing pre-PCV10 with post-PCV10 periods, statistically significant declines were noted for healthy carriers (before 73 [23%] of 313 *vs* after 38 [13%] of 284; p=0·0026), and patients with pneumonia (before 49 [42%] of 116 *vs* after 37 [29%] of 129; p=0·037). No significant difference was noted for invasive pneumococcal disease isolates (before 59 [75%] of 79 *vs* after nine [64%] of 14; p=0·63).

**Figure 1:**
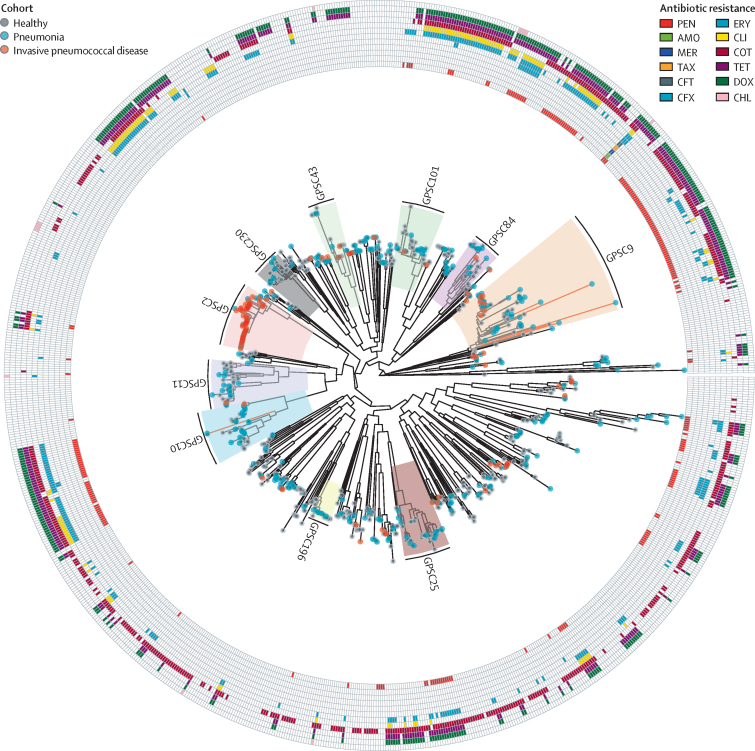
Single nucleotide polymorphism maximum-likelihood phylogeny of the pneumococcal population from Nepalese children The taxa belonging to the largest GPSCs are highlighted. Predominant serotype in each cluster was GPSC2 (serotype 1), GPSC9 (serotype 14), GPSC10 (serotype 10A), GPSC11 (serotype 6A), GPSC25 (serotype 15B), GPSC43 (serotype 9V), GPSC84 (serotype 19A), GPSC101 (serotype 23F), GPSC196 (serotype 11A), and GPSC230 (serotype 34). Red branches are taxa with the same common ancestor as the GPSC, but are not similar enough to be classified within it. The colour of the taxa indicates the source of each isolate; red is sterile site, blue is nasopharynx of child admitted with pneumonia, and grey is nasopharynx of a healthy carrier. The columns represent isolates that possess loci conferring antibiotic resistance. The tree is midpoint rooted. AMO=amoxicillin. CFT=ceftriaxone. CFX=cefuroxime. CHL=chloramphenicol. CLI=clindamycin. COT=cotrimoxazole. DOX=doxycycline. ERY=erythromycin. GPSC=Global Pneumococcal Sequencing clusters. MER=meropenem. PEN=penicillin. TAX=cefotaxime. TET=tetracycline.

912 (97%) of 935 samples were classified into 144 existing GPSCs with the remaining 23 samples classed as novel strains (figure 1; appendix 1 pp 12–14). Some serotypes were only identified within a very restricted range of clusters; eg, serotype 1 was found in three sequence clusters and serotype 14 was found in two sequence clusters (appendix 1 p 15). Conversely, some serotypes were more widely expressed across different clusters; eg, serotypes 6A (13 clusters) and 6B (11 clusters). An interactive version of these data is available online.

**Figure 2:**
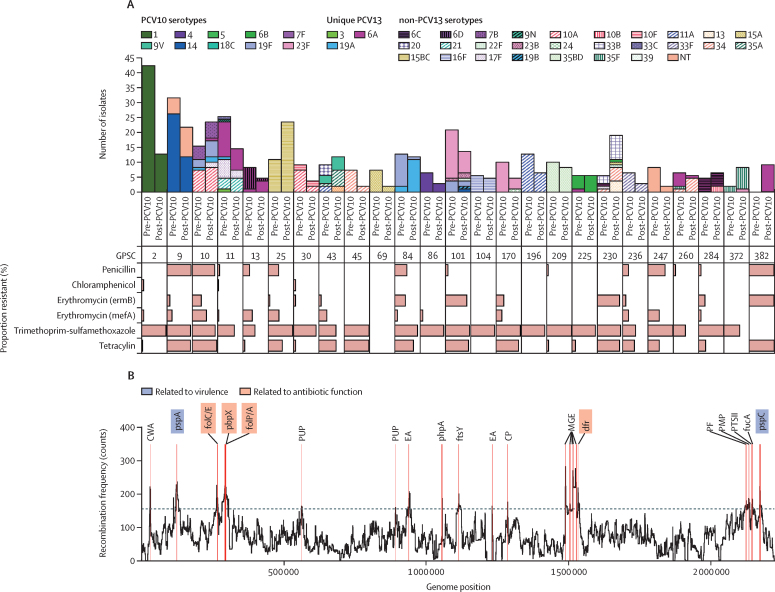
Impact of clinical interventions on pneumococci isolated from Nepalese children (A) Prevalence of PCV10 and PCV13 covered serotypes and percentage of isolates resistant to antibiotics within the GPSCs detected among Nepalese children before (pre-PCV10) and after (post-PCV10) the introduction of PCV10 to the infant immunisation schedule. (B) Hotspots of recombination as determined across the ten most prevalent GPSCs with genes known to relate to virulence through interaction with the human immune system highlighted in blue and known to relate to antibiotic function highlighted in red. CP=CAAX protease (PRIP locus). CWA=cell-wall-binding amidase/autolysin (pseudogene). dfr=dihydrofolate reductase. EA=episilon antitoxin. ftsY=putative signal recognition particle 54. folC/E=dihydrofolate synthase C and E. folP/A=dihydrofolate synthase P and A. fucA=fuculose-1-phosphate aldolase. GPSC=Global Pneumococcal Sequencing cluster. MGE=mobile genetic element. pbpX=penicillin-binding protein 2x. PCV10=ten-valent pneumococcal conjugate vaccine. PCV13=13-valent pneumococcal conjugate vaccine. PF=putative fucosidase. pspA=pneumococcal surface protein A. PMP=putative membrane protein. pspC=pneumococcal surface protein C. PTSII=PTS system IIc component. PUP=putative uncharacterised protein.

121 clusters were identified in the pre-PCV10 period and 92 in the post-PCV10 period. Within the ten largest clusters there were four clusters (GPSCs 2, 9, 84, and 101), which predominantly expressed a PCV10 serotype before vaccine introduction (figure 2). GPSCs 9, 84, and 101 showed an association between PCV10 use and variation in capsular expression with a decrease in PCV10 serotypes and an increase in non-PCV10 serotypes noted when comparing the pre-PCV10 with post-PCV10 periods (appendix 1 p 9). For example, within GPSC84 there was a significant decline in PCV10-covered serotype 19F expression (pre-PCV10 12 of 14 *vs* post-PCV10 one of 13; p<0·0001) and a significant increase in non-PCV10 covered serotype 19A expression (pre-PCV10 two of 14 *vs* post-PCV10 12 of 13, p<0·0001). The most recent common ancestor for serotypes 19A and 19F within GPSC84 is estimated to have been in 1989 (appendix 1 p 16).

1969 antibiotic resistance loci were detected across the 935 samples and 631 (68%) of 935 of the isolates had at least one antibiotic resistance locus detected (figure 1). The most prevalent loci were those conferring resistance to co-trimoxazole (506 [54%] of 935). Additionally, co-trimoxazole was the most prevalent resistance loci in every cohort for each pre-PCV10 and post-PCV10 period. The proportion of isolates resistant to penicillin, cefuroxime, erythromycin, clindamycin, and tetracycline significantly increased from the pre-PCV10 to post-PCV10 periods (table; appendix 1 p 10). The highest prevalence of penicillin resistance was found among GPSCs 9, 10, and 382 (figure 2). No significant declines in prevalence of antibiotic resistance were observed when comparing pre-PCV10 with post-PCV10 periods.

**Table:** Antibiotic resistance (non-susceptibility) detected among pneumococci isolated from Nepalese children before and after PCV10 introduction

	**Isolates collected pre-PCV10 (n=508)**		**Isolates collected post-PCV10 (n=427)**		**p value**
	Non-susceptible isolates, n (%)	Susceptible isolates, n (%)	Non-susceptible isolates	Susceptible isolates, n (%)	
Penicillin	76 (15%)	432 (85%)	138 (32%)	289 (68%)	<0·0001
Amoxicillin	0	508 (100%)	3 (1%)	424 (99%)	0·19
Cefuroxime	15 (3%)	493 (97%)	37 (9%)	390 (91%)	0·0003
Ceftriaxone	6 (1%)	502 (99%)	7 (2%)	420 (98%)	0·75
Chloramphenicol	4 (1%)	504 (99%)	9 (2%)	418 (98%)	0·15
Clindamycin	37 (7%)	471 (93%)	110 (26%)	317 (74%)	<0·0001
Co-trimoxazole	397 (78%)	111 (22%)	347 (81%)	80 (19%)	0·27
Erythromycin	79 (16%)	429 (84%)	196 (46%)	231 (54%)	<0·0001
Meropenem	2 (<1%)	506 (99%)	7 (2%)	420 (98%)	0·11
Tetracycline	172 (34%)	336 (66%)	212 (50%)	215 (50%)	<0·0001

Isolates are from 2005 to 2018; programmatic PCV10 was introduced in Nepal in 2015. PCV10=ten-valent pneumococcal conjugate vaccine.

Regions of recombination across the ten most prevalent clusters were used to ascertain which genes were under evolutionary pressure. The amount of recombination detected across each GPSC was variable, as exemplified by the recombination to mutation ratio of GPSC2 (3·2) compared with GPSC9 (13; appendix 1 p 11). Combining the recombination region data for all ten clusters demonstrated that the sites of recombination were non-random and that the most common regions of recombination included virulence factors (*pspA* and *pspC*) and loci of antibiotic targets (*pbpX, folA, folC, folE*, and *folP*; figure 2).

Non-typeable isolates, which are not targeted by any PCV, were recovered from healthy individuals (n=30), patients with pneumonia (n=30), and one patient with invasive pneumococcal disease. Non-typeable isolates were mainly represented by the genetic backgrounds of GPSC9 (n=17), GPSC247 (n=11), GPSC148 (n=9), and GPSC60 (n=7). Seven different genetic configurations of the cps were identified, with most non-typeable isolates (43 [70%] of 61) found to have *pspC* within the cps region (appendix 1 p 17). *pspC* containing cps loci were predominantly identified in GPSC9 (15 [35%] of 43), GPSC148 (nine of 43), and GPSC247 (eight [19%] of 43; appendix 1 p 18). Non-*pspC* containing cps were predominantly identified in GPSC60 (appendix 1 p 18).

Unlike GPSC148 and GPSC247, GPSC9 has both encapsulated (serotype 14) and non-typeable pneumococcal isolates. To place this strain into a global context, a global phylogeny of GPSC9 was constructed using 748 isolates from 31 countries, including serotype 14 (n=42) and non-typeable (n=12) isolates from Nepal. Nepalese isolates were not clustered together, suggesting multiple importations of GPSC9 into the country, given the mutation rate within this strain (appendix 1 p 18). Recombination analysis also indicates independent acquisition of the *pspC*-containing cps region in non-typeable isolates (appendix 1 p 18).

**Figure 3:**
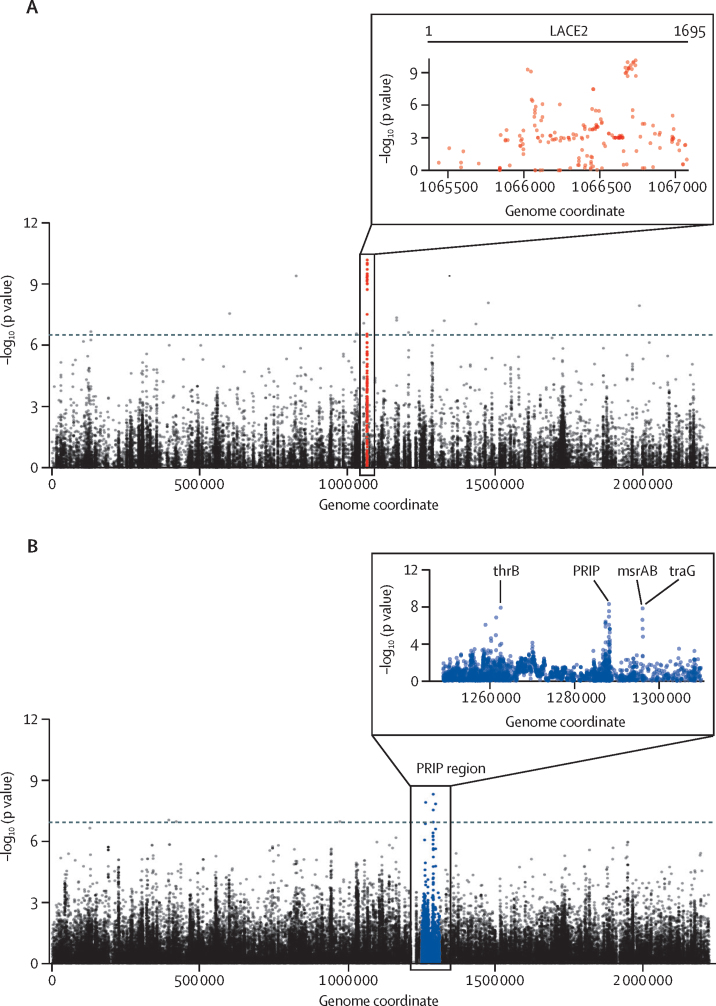
Manhattan plots of genome-wide association analyses (A) Comparison of pneumococcal isolates from children with pneumonia with healthy children identified variants within the *lacE2* locus to have the strongest association with pneumonia isolates. (B) Comparison of children with invasive pneumococcal disease with those from healthy community-based children identified variants within the PRIP region to be associated with disease. Closer inspection of the PRIP region identified these variants of interest to reside within thrB, PRIP, msrAB, and traG.

Pneumococcal genetic variants associated with disease were identified by conducting genome-wide association analyses. A GWAS comparing nasopharyngeal isolates from children with pneumonia (cases) with those from healthy community-based children (controls) identified variants within the *lacE2* to have a strong association with disease (figure 3). The lead SNP was located at position 1311, with supporting SNPs spanning a region between positions 1258 and 1312, associated with a region of deletion in many isolates. The phylogeny of *lacE2* demonstrated two distinct clusters (appendix 1 p 19). Pneumonia isolates were significantly more likely to be found in cluster one (189 of 577) than cluster two (56 of 265) of *lacE2* (p=0·0006). Examination of the sequence of *lacE2* demonstrated uniform regions of deletion in many of the cluster one isolates. Invasive pneumococcal disease isolates had these regions deleted more frequently when compared with pneumonia and healthy carriage isolates, as exemplified by the proportion of isolates with a deletion at *lacE2* spanning base pairs 486–621 (invasive pneumococcal disease 82 of 93 *vs* pneumonia 189 of 245, p=0·034; and invasive pneumococcal disease 82 of 93 *vs* healthy children 390 of 597, p<0·0001; appendix 1 p 20).

A further GWAS comparing the invasive pneumococcal disease isolates with those from healthy children demonstrated variants in *PRIP* to have the strongest association with invasive pneumococcal disease (figure 3). Proximal to *PRIP* we also identified variants of interest within *traG* and an intergenic region between *msrAB* and *thrB*. Examination of *PRIP* showed that 592 isolates had less than 50% sequencing coverage (resulting in a smaller phylogeny of 343 isolates), consistent with deletion or recombination, or both, of the region (appendix 1 p 21). Significantly more isolates from healthy carriers had the *PRIP* deleted (416 of 597) compared with invasive pneumococcal disease (38 of 93; p<0·0001) and pneumonia isolates (138 of 245; p=0·0003).

A hotspot of recombination identified in this study resided within the PRIP region. Comparison of the region downstream of *PRIP* between representative isolates from GPSC2, 9, and 11, indicated GPSC2 to have a conserved PRIP region, GPSC9 to have deletions within the CAAX protease, and GPSC11 to have deletions across all genes except for putative uncharacterised protein 3 (PUP3; appendix 1 p 22).

## Discussion

To our knowledge, this is the first study to examine the molecular epidemiology of pneumococci isolated from Nepalese children. We show evidence that the introduction of PCV10, antibiotic use, and host–pathogen interaction are key effectors of genetic variation in this population. We also show that variants within *lacE2* and *PRIP* are associated with pneumonia and invasive pneumococcal disease, respectively.

The reduced prevalence of capsular loci encoding PCV10 serotypes and the serotype shift to non-PCV10 serotypes within lineages in this study is consistent with earlier studies examining the effect of programmatic PCV introduction on pneumococcal lineages in other settings.^5^ Close monitoring of GPSC9 will be particularly important in this population because it is frequently isolated from children with pneumonia, is resistant to multiple first-line antibiotics, and is capable of evading vaccines through loss of capsular expression. The loss of capsular expression within GPSC9 isolates appears to be associated with acquisition of the *pspC* virulence factor (binds IgA1 and complement factor H, part of the adaptive and innate host immune responses, respectively) within the cps locus.

Community-based pharmacies are the primary antibiotic access points in Nepal, with up to 97% of these stores dispensing to the public without a prescription.^23^ Guidance on antibiotic use for childhood pneumonia by the Nepalese Ministry of Health and Population has generally been in keeping with WHO recommendations, with the most notable change occurring in 2014, when first-line treatment was changed from co-trimoxazole to amoxicillin.^24^ Resistance patterns shown both in this study and others appear to reflect these policies, with low rates of pneumococcal penicillin resistance and high levels of co-trimoxazole resistance before 2014.^25^ We further demonstrate that there is ongoing widespread resistance to co-trimoxazole with rising prevalence of penicillin and erythromycin resistance. These antibiotic resistance patterns suggest there is a possibility that a child admitted with pneumococcal pneumonia in this setting might be infected with a resistant bacterial strain, and second-line treatments such as third-generation cephalosporins should be considered early if there is a poor response to initial therapy or a history of antibiotic use before admission. Further policies to regulate community-based antibiotic sources should also be considered.

Recombination hotspots provide an insight into which genetic regions are under positive selection pressure, with a large study of carriage samples from the Maela community on the Thai–Burmese border, conducted before PCV introduction, showing hotspots of recombination within genes of antibiotic targets and pneumococcal surface proteins.^6^ We similarly show that antibiotic targets such as the penicillin-binding proteins and the enzymes of the dihydrofolate reductase pathway are common regions of recombination in the Nepalese population. We also show that the genes encoding virulence factors PspC and PspA (binds lactoferrin and inhibits complement activity, inhibiting the host's innate immune response) are under selective pressure. This is consistent with immune selection after natural infection and is supported by serum studies showing antibody development to strain-specific variations of PspC and PspA.^26^ These findings coupled with earlier studies in animal challenge models that have shown PspC and PspA have a key role in colonisation and disease progression, rationalise use of these proteins as vaccine candidates.^27^

We showed that variants within *lacE2*, which has a role in pneumococcal nutrient uptake, were strongly associated with carriage isolates from children with pneumonia compared with those from healthy community-based children. A murine model of pneumococcal disease demonstrated that *lacE2* expression correlated with biofilm formation and disease-causing capacity of pneumococci.^28^ However, a study examining the within-host variations that occur during colonisation identified parallel variations to frequently occur in *lacE2*, suggesting adaptation of this locus occurs in response to colonisation.^29^ Identification of this variant using rapid molecular diagnostics might assist in clinical decision making. Drugs targeting the phosphoenolpyruvate PTS, of which *lacE2* is a component, might be a potential avenue for clinical intervention, with pyrimidinedione being shown to inhibit pneumococcal biofilm formation in vitro.^30^

We also showed that variants within *PRIP* were associated with invasive pneumococcal disease and that carriage isolates from healthy children were more likely to have this gene and a group of genes downstream of it deleted when compared with isolates from children with pneumonia or invasive pneumococcal disease. Most of the genes downstream of *PRIP* are yet to be characterised; however, earlier studies suggest that the most distal gene, a CAAX protease, possibly has a bactericidal role. We hypothesise that this group of proteins might assist in rapid colonisation through an antibacterial mechanism that eliminate or reduces the presence of competitive bacteria in niches within the respiratory mucosa. Future interventions inhibiting this mechanism might reduce the invasive disease potential of strains with this genetic factor.

Some limitations of this study include the duration of the post-PCV10 period from which samples were collected and the small sample size of post-PCV10 invasive pneumococcal disease isolates. Identification of additional invasive pneumococcal disease isolates from the post-PCV10 period should be a priority, but is challenging because the incidence of invasive pneumococcal disease is expected to be much lower due to vaccine use. Confirmation of GWAS findings in other datasets would also strengthen the findings described in this study.

We conclude that PCV10 introduction is associated with a reduction in vaccine serotype expression across this pneumococcal population; however, the strains that previously expressed PCV10 serotypes are still detectable. Judicious use of antibiotics in this population should be a key policy directive because, as this study showed, many hotspots of recombination in this population are reflective of adaptation to antibiotics. Ongoing observation of this population as the PCV10 vaccination programme matures, and co-trimoxazole use is phased out, will provide further insight into pneumococcal adaptation to these clinical interventions. Finally, we identified variants within the pneumococcal genome that were associated with disease. Confirmation of these findings in similar populations and further detailed exploration of the biological roles of these genes in the context of pneumococcal disease could lead to the development of innovative diagnostic, therapeutic, or preventive tools.

## Data sharing

All isolate assembly data were deposited in the European Nucleotide Archive (appendix 2).

## Declaration of interests

RK receives a National Health and Medical Research Council (NHMRC) Emerging Leader Fellowship (GNT1174010). MJC is a National Institute for Health Research (NIHR) Academic Clinical Lecturer and was funded by a Wellcome Trust Clinical Fellowship (104439/Z/14/Z) for part of this work. AJP is Chair of UK Department of Health and Social Care's (DHSC) Joint Committee on Vaccination & Immunisation (JCVI). He is a member of WHO's Strategic Advisory Group of Experts. AJP is an NIHR Senior Investigator. The views expressed in this Article do not necessarily represent the views of DHSC, JCVI, NIHR, or WHO. AJP is chief investigator on clinical trials of Oxford University's COVID-19 vaccine funded by NIHR. Oxford University has entered a joint COVID-19 vaccine development partnership with AstraZeneca.
