# Supplementary material for: Effect of childhood vaccination and antibiotic use on pneumococcal populations and genome-wide associations with disease among children in Nepal: an observational study
Source: Lancet Microbe. 2022 Jul;3(7):e503–11. doi: 10.1016/S2666-5247(22)00066-0 (PMC9242864; doi:10.1016/S2666-5247(22)00066-0)
Supplement: Supplementary appendix 1 [file mmc1.pdf]

THE LANCET
Microbe

Supplementary appendix

This appendix formed part of the original submission and has been peer reviewed. We post it as supplied by the authors.

Supplement to: Kandasamy R, Lo S, Gurung M, et al. Effect of childhood vaccination and antibiotic use on pneumococcal populations and genome-wide associations with disease among children in Nepal: an observational study. *Lancet Microbe* 2022; published online May 9 . https://doi.org/10.1016/S2666-5247(22)00066-0.

APPENDIX

SUPPLEMENTARY METHODS ... 2

SUPPLEMENTARY TABLES ... 3

Supplementary table 1. Characteristics of pneumococcal samples from Nepalese children that underwent whole-genome sequencing. ... 3

Supplementary table 2. Sub-group characteristics of pneumococcal samples from healthy Nepalese children that underwent whole-genome sequencing. ... 4

Supplementary table 3. Clinical characteristics of children admitted with pneumonia ... 5

Supplementary table 4: Paediatric antibiotic usage patterns at Patan Hospital, Kathmandu, Nepal ... 6

Supplementary table 5. Serotypes identified among Nepalese children according to cohort and vaccine period ... 7

Supplementary table 6. PCV coverage of sequenced isolates from Nepalese children. ... 8

Supplementary table 7. PCV10 coverage of the ten largest GPSCs pre- and post-PCV10 introduction. ... 9

Supplementary table 8. Antibiotic resistance (non-susceptibility) detected among pneumococci isolated from healthy Nepalese children before and after PCV10 introduction. ... 10

Supplementary table 9. Recombination to mutation ratio of the 10 largest GPSCs within the Nepalese cohort. ... 11

SUPPLEMENTARY FIGURES ... 12

Supplementary figure 1. Maximum likelihood SNP phylogeny of pneumococcal isolates from the nasopharynx of healthy community-based Nepalese children. ... 12

Supplementary figure 2. Maximum likelihood SNP phylogeny of pneumococcal isolates from the nasopharynx of Nepalese children admitted to hospital with pneumonia. ... 13

Supplementary figure 3. Maximum likelihood SNP phylogeny of pneumococcal isolates from sterile sites of Nepalese children with invasive pneumococcal disease. ... 14

Supplementary figure 4. The most prevalent serotypes and the number of GPSCs each serotype was identified within ... 15

Supplementary figure 5. Temporal phylogeny of GPS cluster 84 demonstrating the most-recent common ancestor of serotypes 19A and 19F occurred in 1989 (before PCV10 introduction). ... 16

Supplementary figure 6. A maximum likelihood tree of 935 pneumococcal isolates from Nepal, 2005-2018. The non-typeable isolates were highlighted and coloured by Global Pneumococcal Sequence Clusters (GPSCs) and their corresponding cps genetic configuration are shown in the metadata block. ... 17

Supplementary figure 7. The genetic configuration of cps loci in non-encapsulated isolates. ... 18

Supplementary figure 8. Maximum likelihood phylogeny of lacE2, and clustering (green shading=cluster 1 and red shading =cluster 2), demonstrates two distinct clades and the corresponding nucleotide sequence of each sample was aligned and shown in the right panel, A=green, C=red, G=blue, T=yellow, and N=white.. 19

Supplementary figure 9. Proportion of isolates within each cohort with a deletion at each base pair within *lacE2*. ... 20

Supplementary figure 10. Maximum likelihood phylogeny of PRIP after filtering for sequences with greater than 50% coverage, N= 343 isolates, and the corresponding nucleotide sequence of each sample was aligned and shown in the right panel, A=green, C=red, G=blue, T=yellow, and N=white. ... 21

Supplementary figure 11. Comparison between representative GPSC isolates of the genes downstream of PRIP ... 22

SUPPLEMENTARY METHODS

Whole genome sequencing

To ensure high-quality sequence in downstream analysis, only samples with these criteria were included: less than 500 contigs, mapped to >60 % of *Streptococcus pneumoniae* ATCC 700669, had an assembly length between 1,900,000 - 2,300,000 bp; greater than 20 times coverage, and <15% of heterozygous single nucleotide polymorphism (SNP) of the total SNPs. Selected isolates then underwent Kraken analysis to identify non-pneumococcal species contamination; any isolate with a content of more than 10% from another non-pneumococcal species was excluded. Duplicate runs of the same isolate were also identified and removed.

Assembly creation

BWA,[1] STAMPY,[2] Bowtie2,[3] and Tophat[4] were used to conduct *de novo* assembly of genomes before reads were mapped back to each assembly using SMALT. In this cohort the average number of contigs per sequenced isolate was 42.

Antibiotic resistance loci

Beta-lactam resistance was identified by typing penicillin binding protein (PBP) alleles.[5] blastp was then used to compare typed PBP alleles with the PBP database. Resistance or sensitivity could then be assigned based on the PBP allele identities and previously described correlation with phenotypic antibiotic breakpoints. The Clinical and Laboratory Standards Institute breakpoint references M100-ED28:2018 were used to inform antibiotic resistance using this pipeline. The higher meningitis breakpoint threshold was applied for penicillin and ceftriaxone.

Genome-wide association study

Annotations of each isolate from the selected study cohorts of interest were generated by the Wellcome Sanger Institute annotation pipeline and analysed using ROARY to determine the core genome alignment and gene presence/absence. The population structure was estimated using mash on the isolate assemblies and the distances between pairs of samples calculated. Multi-dimensional scaling (MDS) was performed on the distance values and maximum number of dimensions determined manually. pyseer was then used to run the analysis on the COGs, in this case using a maximum of 10 dimensions. The MDS decomposition from the COG analysis was used to inform pyseer analysis, using a minimum allele frequency of 0.05, on SNPs produced from mapping reads against the reference genome of *Streptococcus pneumoniae* ATCC 700669.

REFERENCES

1. Li H and Durbin R. Fast and accurate short read alignment with Burrows-Wheeler Transform. Bioinformatics, 2009, 25:1754-60.
2. Lunter G and Goodson M. Stampy: A statistical algorithm for sensitive and fast mapping of Illumina sequence reads. Genome Res, 2011 21: 936-939.
3. Langmead B, and Salzberg S. Fast gapped-read alignment with Bowtie 2. Nature Methods, 2012, 9:357-359.
4. Langmead B, Trapnell C, Pop M, Salzberg S. Ultrafast and memory-efficient alignment of short DNA sequences to the human genome. Genome Biology, 2009, 10:R25.
5. Metcalf BJ, Gertz RE, Gladstone RA, *et al.* Strain features and distributions in pneumococci from children with invasive disease before and after 13-valent conjugate vaccine implementation in the USA. *Clin Microbiol Infect* 2016; 22: 60.e9-60.e29

SUPPLEMENTARY TABLES

Supplementary table 1. Characteristics of pneumococcal samples from Nepalese children that underwent whole-genome sequencing.

Pre/Post-PCV10	Cohort	Number of isolates, n (%)	Source	Time period of collection	Age years, mean (range)	Male, %
Pre	Healthy children	313 (34)	Nasopharynx	Feb 2009 - Sep 2014	0.87 (0.08 - 4)	49
Post	Healthy children	284 (30)	Nasopharynx	Nov 2017 - May 2018	1.26 (0.51 - 4.8)	56
Pre	Inpatients with pneumonia	116 (12)	Nasopharynx	Mar 2014 - Apr 2015	2.63 (0 - 13)	64
Post	Inpatients with pneumonia	129 (14)	Nasopharynx	Aug 2015 - May 2018	1.96 (0.17 - 11.3)	53
Pre	Invasive disease	79 (8)	Sterile site	Jan 2005 - May 2015	3.76 (0.17 - 14)	52
Post	Invasive disease	14 (2)	Sterile site	Aug 2015 - Sep 2017	5.96 (0.16 - 10.9)	71
Pre	All	508 (54)	Nasopharynx or sterile site	Jan 2005 - Aug 2015	1.72 (0 - 14)	52
Post	All	427 (46)	Nasopharynx or sterile site	Aug 2015 - May 2018	1.62 (0.17 - 11.3)	55

Supplementary table 2. Sub-group characteristics of pneumococcal samples from healthy Nepalese children that underwent whole-genome sequencing.

Cohort	Number of isolates, n (% of all healthy carriers)	Time period of collection	Age years, mean (range)	Male, %
Healthy children 2009	104 (17)	Feb 2009 - Mar 2009	0.7 (0.08 - 1)	49
Healthy children 2012	86 (14)	May 2012 - Oct 2012	0.71 (0.08 - 1)	47
Healthy children 2014	123 (21)	Apr 2014 - Oct 2014	1.11 (0.41 - 4)	51
Healthy children 2017	138 (23)	Nov 2017 - Dec 2017	1.09 (0.51 - 2)	58
Healthy children 2018	146 (24)	Apr 2018 - May 2018	1.41 (0.72 - 4.8)	53

Supplementary table 3. Clinical characteristics of children admitted with pneumonia

Clinical measure	n/N (%)	no data, N
CRP>60mg/ml	23/61 (38)	184
End-point consolidation on CXR	55/129 (43)	116
Infiltrates on CXR	15/129 (12)	116
Normal CXR	59/129 (46)	116
Blood culture positive for pneumococcus	4/168 (2)	77

Supplementary table 4: Paediatric antibiotic usage patterns at Patan Hospital, Kathmandu, Nepal

	**Fluoroquinolones**
**Pre 2005-2018**	Used for urinary tract infections and dysentery.<br>Also used as second line treatment for enteric fever following azithromycin.
**Pre 2005-2011**	Ofloxacin used for outpatient managed enteric fever.
	**Beta-lactams**
**Pre 2005-2018**	Ampicillin used for children admitted with pneumonia who have had no prior antibiotic treatment.
**Pre 2005-2018**	Ceftriaxone used as second line inpatient treatment of pneumonia or if the patient has had prior antibiotics in the community.
**Pre 2005-2018**	Amoxicillin commonly used in the community for pneumonia over the last two decades
**2010-2018**	Cefixime also used for UTIs, enteric fever, and dysentery
**2010-2018**	Cefixime commonly used in community for pneumonia
	**Co-trimoxazole**
**Pre 2005-2018**	Used for UTI prophylaxis and PCP pneumonia.
**Pre 2005-2014**	Widely used in the community for pneumonia until 2014
	**Macrolides**
**2013-2018**	Azithromycin used for outpatient managed enteric fever (clinically diagnosed).
**Pre 2005-2018**	Azithromycin occasionally used as a single agent for pneumonia.
**Pre 2005-2018**	Azithromycin occasionally used in the community for pneumonia (consistent for last two decades)
	**Tetracyclines**
**Pre 2005-2018**	Occasionally used in older children for cholera and scrub typhus
	**Chloramphenicol**
**Pre 2005-2018**	Occasionally used for children admitted with pneumonia. Rarely used and/or available in the community.

Supplementary table 5. Serotypes identified among Nepalese children according to cohort and vaccine period

Serotype	Healthy pre-PCV10, no. of isolates	Healthy post-PCV10, no. of isolates	Pneumonia pre-PCV10, no. of isolates	Pneumonia post-PCV10, no. of isolates	IPD pre-PCV10, no. of isolates	IPD post-PCV10, no. of isolates
6A	24	23	6	9	1	-
NT	23	19	14	17	3	-
23F	21	10	7	4	5	-
19F	19	6	5	4	1	-
11A	12	11	5	2	-	-
6B	12	10	3	5	3	-
34	11	14	2	3	-	-
6C	11	8	-	2	-	1
35F	11	7	1	3	-	-
14	11	4	11	10	6	-
35B	10	12	3	3	-	-
10A	10	7	3	5	3	-
3	9	7	1	3	1	-
6D	8	-	-	1	-	-
17F	7	5	1	1	-	-
16F	7	4	-	1	-	-
21	7	3	-	2	-	-
15B	6	13	2	2	-	-
15A	6	4	2	1	1	-
20	6	4	-	-	1	-
33F	6	3	1	1	-	-
13	5	9	4	2	-	-
7B	4	6	2	-	-	-
24F	4	5	1	-	-	1
18C	4	2	2	-	1	-
9L	4	1	-	-	-	-
19A	3	13	3	9	1	1
33B	3	7	-	1	-	-
23B	3	4	2	1	1	-
19B	3	4	-	-	-	-
9V	3	4	4	1	3	-
39	3	3	-	1	-	-
9N	3	2	-	-	-	-
8	3	2	2	1	3	-
4	3	1	3	2	1	-
35A	2	7	1	3	1	-
15C	2	6	2	3	-	-
23A	2	4	-	4	-	-
31	2	4	-	1	-	-
22F	2	4	1	1	-	-
24B	2	1	-	-	-	-
35C	2	1	-	-	-	-
12F	2	1	1	-	-	-
18A	2	-	1	1	-	-
38	2	-	-	1	-	-
7C	1	5	-	1	-	-
10F	1	2	1	-	-	-
15F	1	2	-	2	-	-
17A	1	2	-	-	-	-
24A	1	1	-	-	-	-
22A	1	-	-	-	-	-
29	1	-	-	1	-	-
36	1	-	-	-	-	-
10B	-	2	-	1	-	-
24	-	1	-	-	-	-
32A	-	1	-	-	-	-
37	-	1	-	-	-	-
48	-	1	-	-	-	-
7F	-	1	1	1	-	1
16C	-	-	-	1	-	-
42	-	-	1	1	-	-
2	-	-	1	-	2	1
28F	-	-	1	-	-	-
33C	-	-	1	-	-	-
33D	-	-	1	-	-	-
12A	-	-	-	-	1	-
25F	-	-	-	-	1	-
41	-	-	-	-	-	1
5	-	-	1	2	3	1
1	-	-	12	8	36	7

Supplementary table 6. PCV coverage of sequenced isolates from Nepalese children.

Vaccine	Healthy children (n=597), % (no. of isolates)	Pneumonia (n=245), % (no. of isolates)	IPD (n=93), % (no. of isolates)
PCV10	19 (116)	35 (86)	74 (69)
PCV13	33 (195)	48 (117)	78 (73)
PCV15	35 (209)	49 (121)	78 (73)
PCV20	43 (259)	57 (139)	85 (79)
PCV24	47 (280)	58 (142)	89 (83)

PCV10 = serotypes 1, 4, 5, 6B, 7F, 9V, 14, 18C, 19F, and 23F
PCV13 = PCV10 + serotypes 3, 6A, and 19A
PCV15 = PCV13 + serotypes 22F, 23F, and 33F
PCV20 = PCV13 + serotypes 8, 10A, 11A, 12F, 15B, 22F, and 33F
PCV24 = 20 + serotypes 2, 9N, 17F, and 20

Supplementary table 7. PCV10 coverage of the ten largest GPSCs pre- and post-PCV10 introduction.

GPSC	PCV10 coverage	Pre-/ Post-PCV10	Number of isolates
2	NVT	Pre	0
2	PCV10	Pre	47
2	NVT	Post	0
2	PCV10	Post	14
9	NVT	Pre	6
9	PCV10	Pre	29
9	NVT	Post	9
9	PCV10	Post	13
10	NVT	Pre	14
10	PCV10	Pre	3
10	NVT	Post	21
10	PCV10	Post	5
11	NVT	Pre	28
11	PCV10	Pre	0
11	NVT	Post	14
11	PCV10	Post	0
25	NVT	Pre	12
25	PCV10	Pre	0
25	NVT	Post	26
25	PCV10	Post	0
43	NVT	Pre	7
43	PCV10	Pre	3
43	NVT	Post	8
43	PCV10	Post	5
84	NVT	Pre	2
84	PCV10	Pre	12
84	NVT	Post	12
84	PCV10	Post	1
101	NVT	Pre	1
101	PCV10	Pre	22
101	NVT	Post	4
101	PCV10	Post	11
196	NVT	Pre	14
196	PCV10	Pre	0
196	NVT	Post	7
196	PCV10	Post	0
230	NVT	Pre	6
230	PCV10	Pre	0
230	NVT	Post	21
230	PCV10	Post	0

Supplementary table 8. Antibiotic resistance (non-susceptibility) detected among pneumococci isolated from healthy Nepalese children before and after PCV10 introduction.

Antibiotic	Healthy children, pre-PCV10, N non-susceptible/N susceptible (%)	Pneumonia, pre-PCV10, N non-susceptible/N susceptible (%)	Invasive disease, pre-PCV10, N non-susceptible/N susceptible (%)	Healthy children, post-PCV10, N non-susceptible/N susceptible (%)	Pneumonia, post-PCV10, N non-susceptible/N susceptible (%)	Invasive disease, post-PCV10, N non-susceptible/N susceptible (%)
Penicillin	35/278 (11.2)	34/82 (29.3)	7/72 (8.9)	83/201 (29.2)	54/75 (41.9)	1/13 (7.1)
Amoxicillin	0/313 (0)	0/116 (0)	0/79 (0)	0/284 (0)	1/128 (0.8)	1/13 (7.1)
Meropenem	0/313 (0)	0/116 (0)	0/79 (0)	0/284 (0)	1/128 (0.8	1/13 (7.1)
Ceftriaxone	0/313 (0)	0/116 (0)	0/79 (0)	0/284 (0)	1/128 (0.8)	1/13 (7.1)
Cefuroxime	4/309 (1.3)	4/112 (3.4)	0/79 (0)	20/264 (7)	9/120 (7)	1/13 (7.1)
Erythromycin	50/263 (16)	22/94 (19)	7/72 (8.9)	130/154 (45.8)	62/67 (48.1)	4/10 (28.6)
Clindamycin	30/283 (9.6)	6/110 (5.7)	1/78 (1.3)	80/204 (28.2)	28/101 (21.7)	2/12 (14.3)
Co-trimoxazole	160/153 (51.1)	69/47 (59.5)	28/51 (35.4)	171/113 (60.2)	74/55 (57.4)	4/10 (28.6)
Tetracycline	105/208 (33.5)	48/68 (41.4)	19/60 (24.1)	145/139 (51)	65/64 (50.4)	2/12 (14.3)
Chloramphenicol	1/312 (0.3)	0/116 (0)	3/76 (3.8)	5/279 (1.8)	3/126 (2.3)	1/13 (7.1)

Supplementary table 9. Recombination to mutation ratio of the 10 largest GPSCs within the Nepalese cohort.

GPSC	r/m
2	3.2
9	13
10	6.8
11	10.4
25	8.6
43	3.2
84	10.2
101	14.2
196	4.4
230	16.2

SUPPLEMENTARY FIGURES

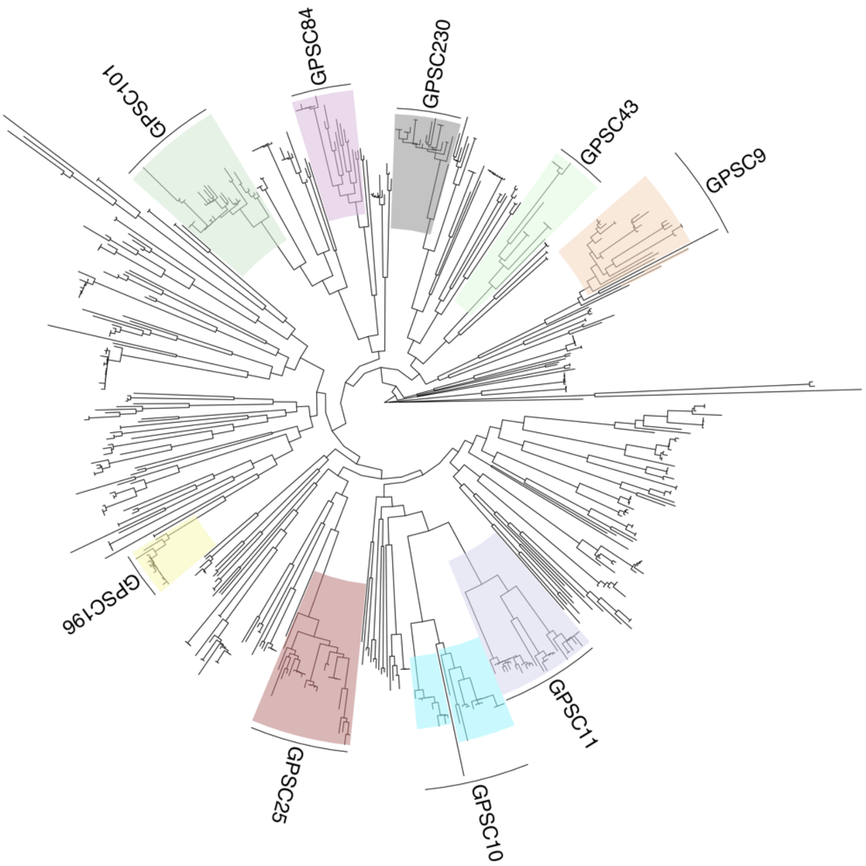

Supplementary figure 1. Maximum likelihood SNP phylogeny of pneumococcal isolates from the nasopharynx of healthy community-based Nepalese children. The largest GPSCs are highlighted.

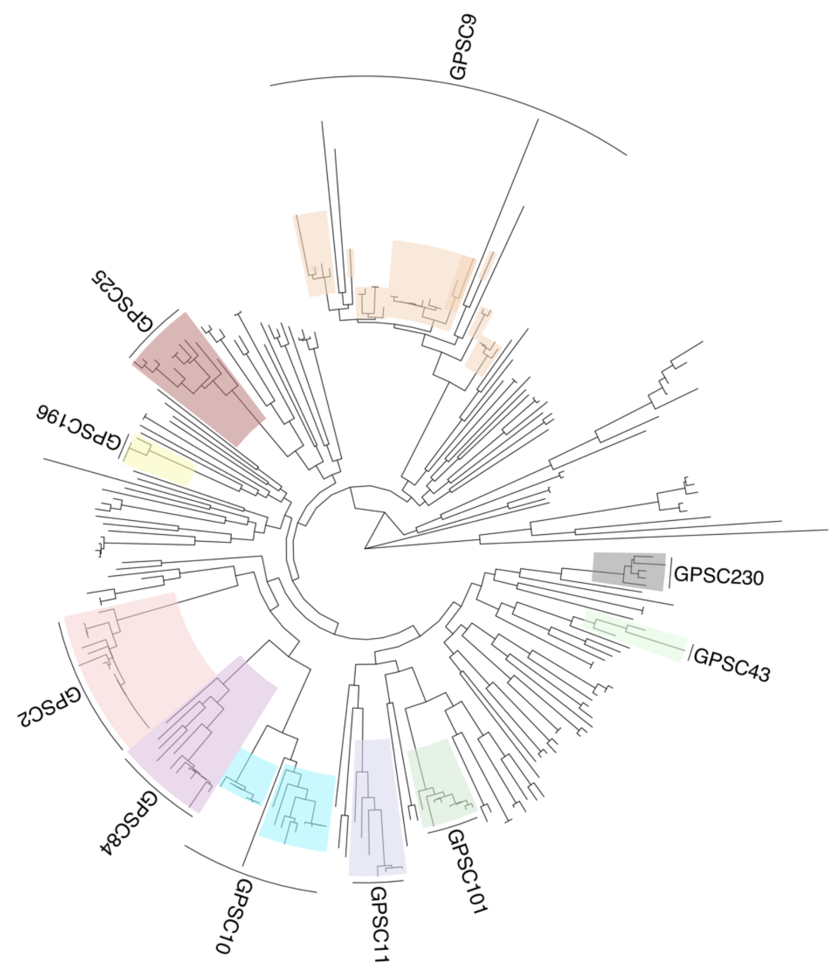

Supplementary figure 2. Maximum likelihood SNP phylogeny of pneumococcal isolates from the nasopharynx of Nepalese children admitted to hospital with pneumonia. The largest GPSCs are highlighted.

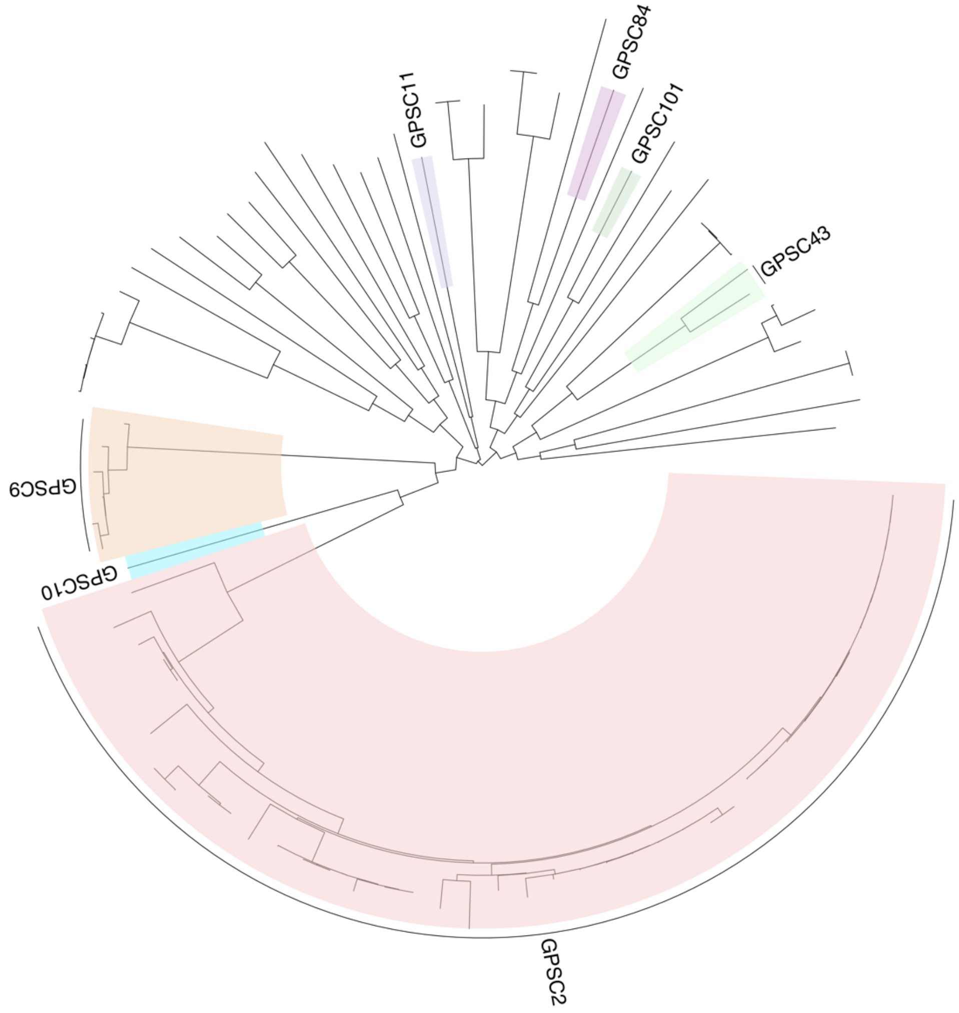

Supplementary figure 3. Maximum likelihood SNP phylogeny of pneumococcal isolates from sterile sites of Nepalese children with invasive pneumococcal disease. The largest GPSCs are highlighted.

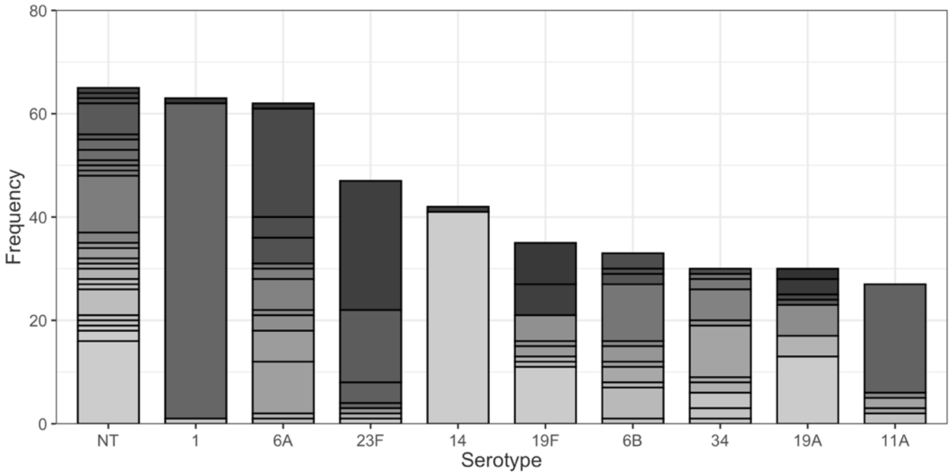

Supplementary figure 4. The most prevalent serotypes and the number of GPSCs each serotype was identified within. NT (25), 1 (3), 6A (13), 23F (7), 14 (2), 19F (8), 6B (11), 34 (11), 19A (6), and 11A (5). NT = non-typeable.

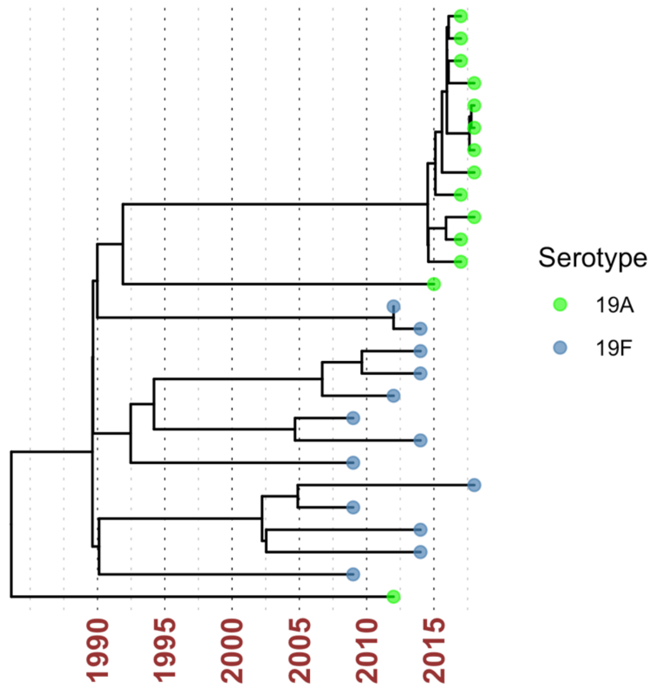

Supplementary figure 5. Temporal phylogeny of GPS cluster 84 demonstrating the most-recent common ancestor of serotypes 19A and 19F occurred in 1989 (before PCV10 introduction).

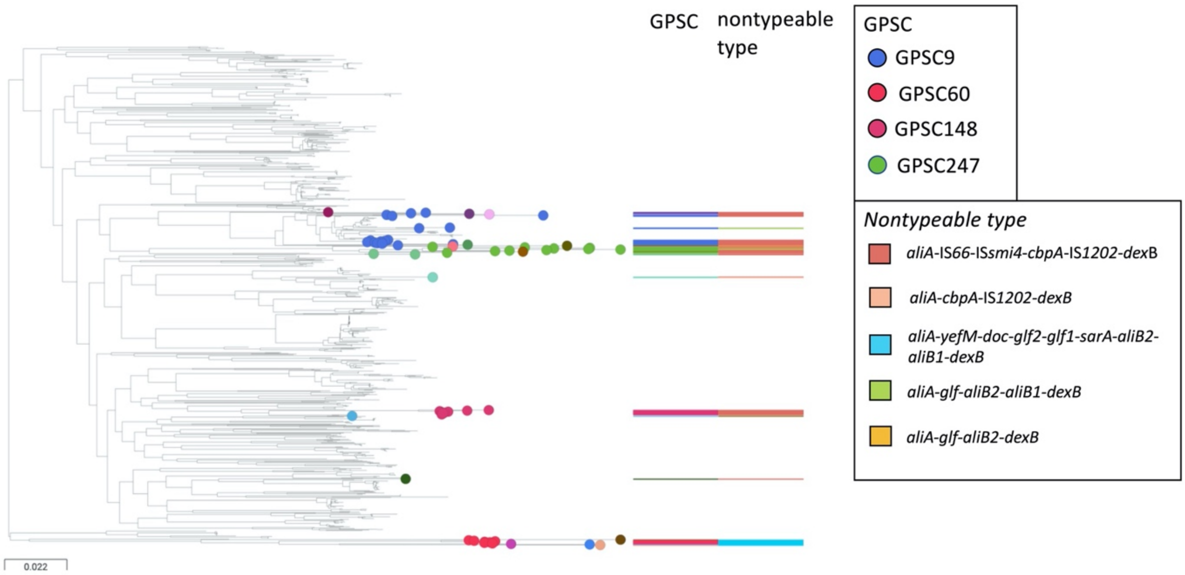

Supplementary figure 6. A maximum likelihood tree of 935 pneumococcal isolates from Nepal, 2005-2018. The non-typeable isolates were highlighted and coloured by Global Pneumococcal Sequence Clusters (GPSCs) and their corresponding cps genetic configuration are shown in the metadata block. The cps genetic configuration of *aliA*-IS*66*-IS*smi4*-*cbpA*-IS*1202*-*dex*B is present in GPSC9, GPSC148 and GPSC247 while *aliA-yefM-doc-glf2-glf1-sarA-aliB2-aliB1-dexB* in GPSC60. This figure can be visualized at https://microreact.org/project/GPS_Nepal/e220f6c1

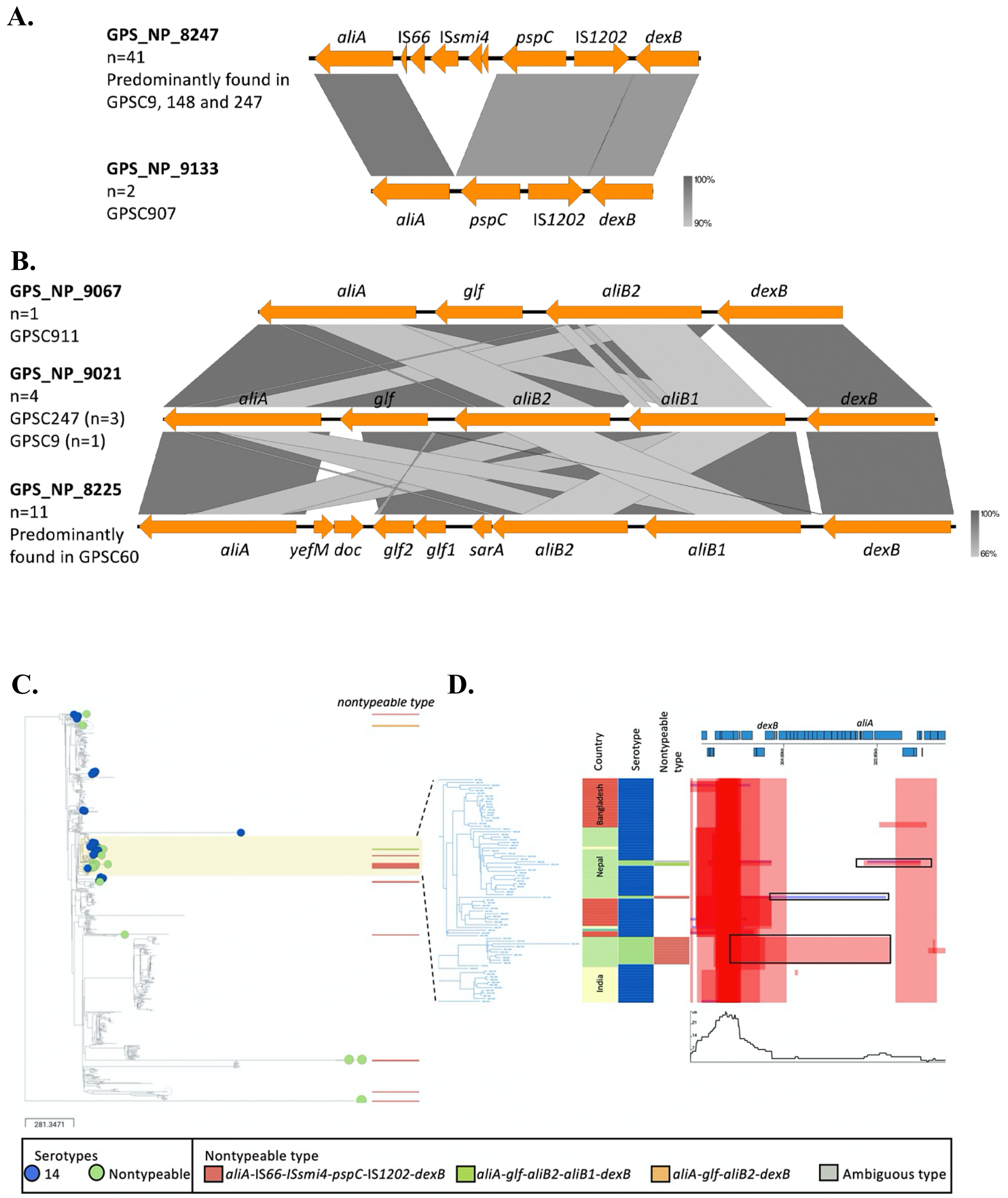

Supplementary figure 7. The genetic configuration of cps loci in non-encapsulated isolates. (A) Cps loci containing *pspC* and (B) without. (C) Global phylogeny of GPSC9 (n=748) from 31 countries and the nodes of 59 pneumococcal isolates from Nepal were coloured by serotypes, indicating multiple importations of GPSC9 into Nepal and *pspC* containing cps acquired multiple times within this lineage. (D) An example of capsular switching from vaccine serotype 14 to non-typeable in a GPSC9 clade from Nepal. Recombinations that occurred within the cps between *dexB* and *aliA* are boxed.

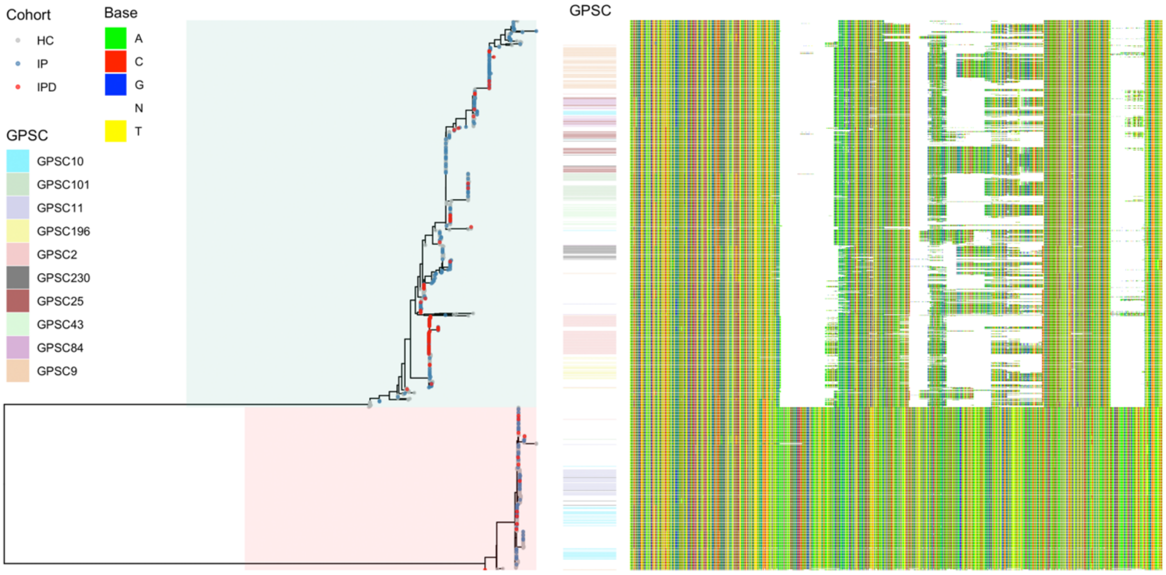

Supplementary figure 8. Maximum likelihood phylogeny of lacE2, and clustering (green shading=cluster 1 and red shading =cluster 2), demonstrates two distinct clades and the corresponding nucleotide sequence of each sample was aligned and shown in the right panel, A=green, C=red, G=blue, T=yellow, and N=white. The lead variants resided within a region that correlated with deletion between bp 1258 and 1312 of *lacE2*.

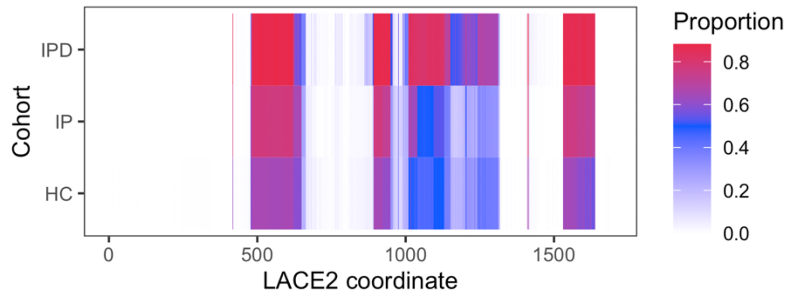

Supplementary figure 9. Proportion of isolates within each cohort with a deletion at each base pair within *lacE2*.

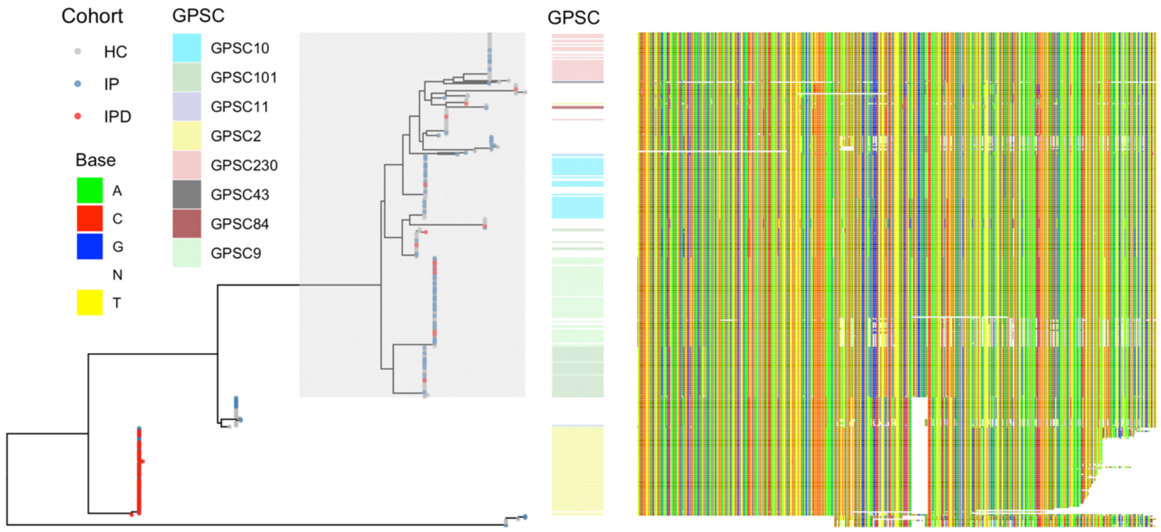

Supplementary figure 10. Maximum likelihood phylogeny of PRIP after filtering for sequences with greater than 50% coverage, N= 343 isolates, and the corresponding nucleotide sequence of each sample was aligned and shown in the right panel, A=green, C=red, G=blue, T=yellow, and N=white. Clustering of *PRIP* demonstrated a single clade shaded in grey, and outliers with limited coverage (unshaded taxa).

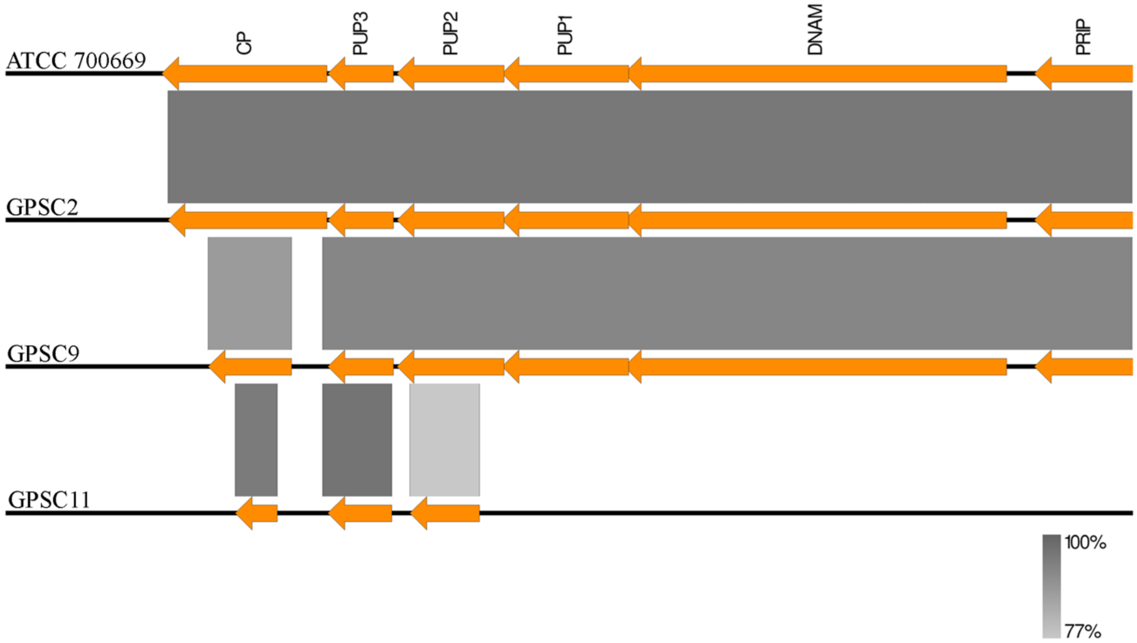

Supplementary figure 11. Comparison between representative GPSC isolates of the genes downstream of PRIP. ATCC 700669 = reference strain Streptococcus pneumoniae ATCC 700669. PRIP=putative replication initiator protein, DNAM=DNA methylase, PUP1=putative uncharacterised protein 1, PUP2=putative uncharacterised protein 2, PUP3=putative uncharacterised protein 3, and CP=CAAX protease.
